# Supplementary material for: The role of interdecadal climate oscillations in driving Arctic atmospheric river trends
Source: Nat Commun. 2024 Mar 8;15:2135. doi: 10.1038/s41467-024-45159-5 (PMC10923828; doi:10.1038/s41467-024-45159-5)
Supplement: Supplementary file 1 — Supplementary Information [file 41467_2024_45159_MOESM1_ESM.pdf]

The Role of Interdecadal Climate Oscillations in Driving Arctic Atmospheric River Trends

Weiming Ma[1], Hailong Wang[1], Gang Chen[2], L. Ruby Leung[1], Jian Lu[1], Philip J. Rasch[3], Qiang Fu[3], Ben Kravitz[1,4], Yufei Zou[1], John J. Cassano[5,6,7], Wieslaw Maslowski[8]

[1]Atmospheric, Climate, and Earth Sciences Division, Pacific Northwest National Laboratory, Richland, WA, USA

[2]Department of Atmospheric and Oceanic Sciences, University of California Los Angeles, Los Angeles, CA, USA

[3]Department of Atmospheric Sciences, University of Washington, Seattle, WA, USA

[4]Department of Earth and Atmospheric Sciences, Indiana University, Bloomington, IN, USA

[5]Cooperative Institute for Research in Environmental Sciences, University of Colorado, Boulder, CO, USA

[6]National Snow and Ice Data Center, University of Colorado, Boulder, CO, USA

[7]Department of Atmospheric and Oceanic Sciences, University of Colorado, Boulder, CO, USA

[8]Department of Oceanography, Naval Postgraduate School, Monterey, California, USA

Corresponding authors: Weiming Ma (weiming.ma@pnnl.gov), Hailong Wang (Hailong.Wang@pnnl.gov)

Supplementary Information:

Supplementary Figures 1-14

Supplementary Table 1

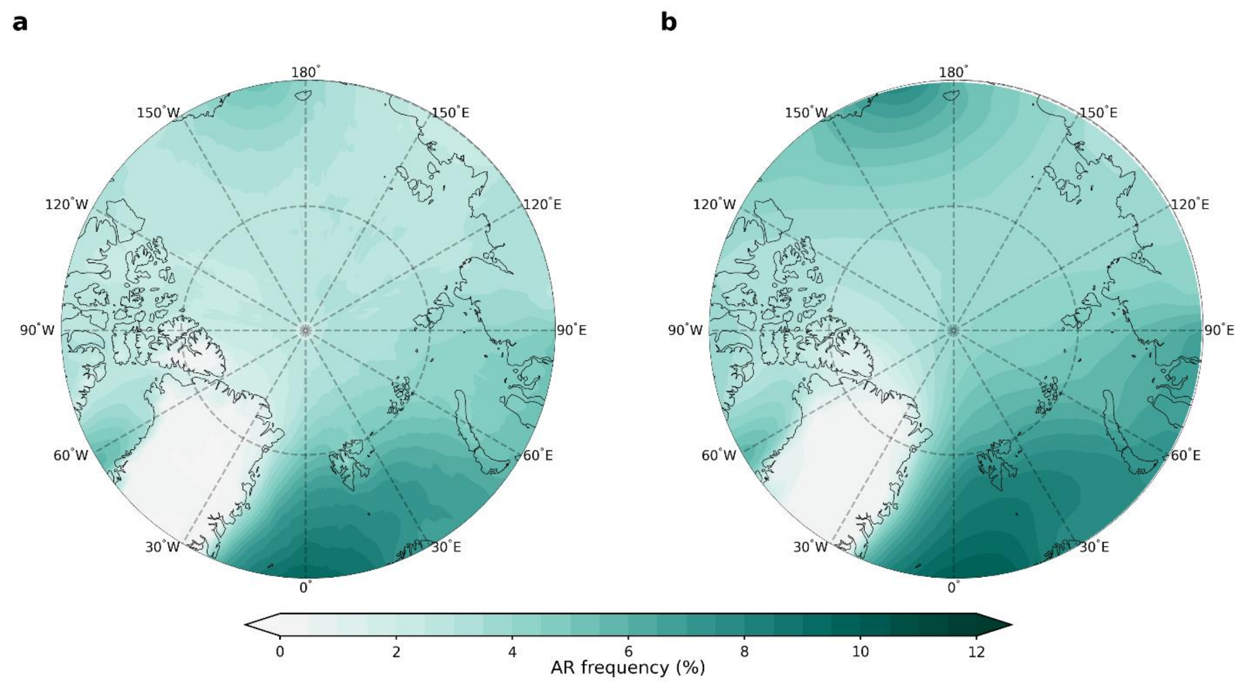

Supplementary Fig. 1 Atmospheric river (AR) climatology. Annual mean AR frequency (1981–2021) over the Arctic in ERA5 (**a**) and LENS2 (**b**).

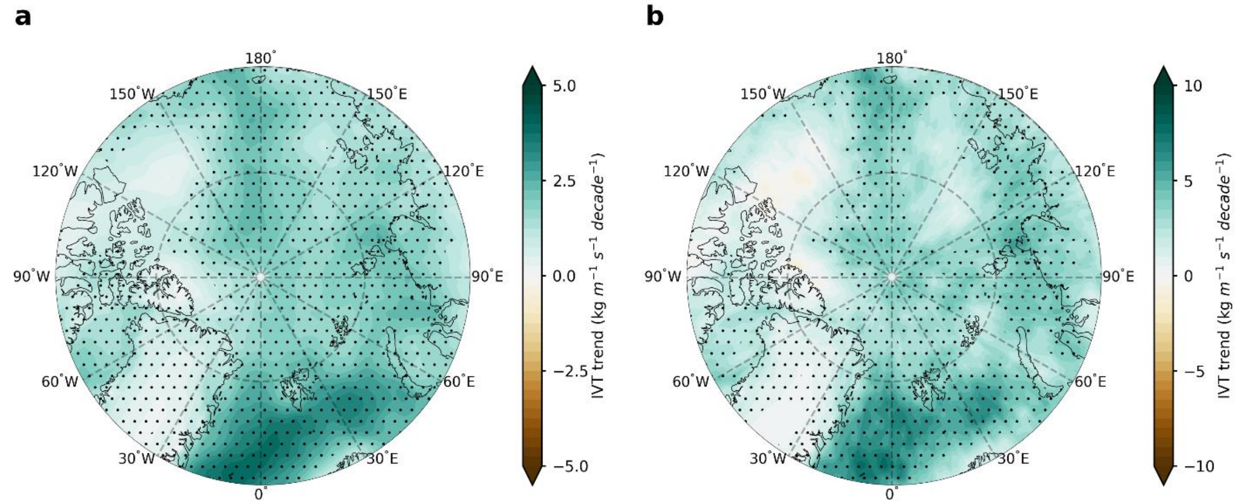

Supplementary Fig. 2 Integrated water vapor transport (IVT) trends in ERA5. Annual mean IVT trends (a) and the annual 85th percentile IVT magnitude trends over the Arctic from 1981 to 2021. Stippled areas indicate trends are significant at the 0.05 level based on the Student's t-test.

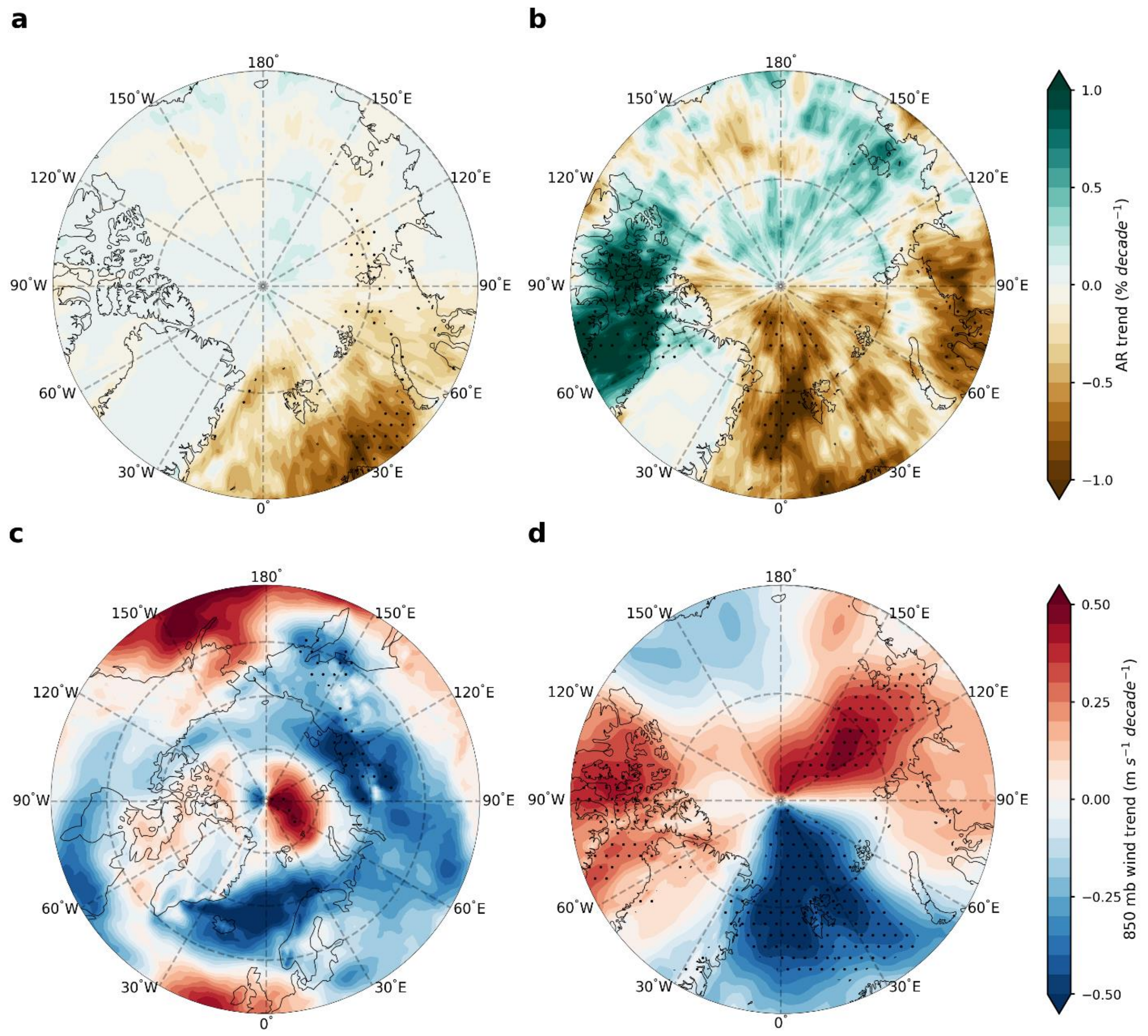

Supplementary Fig. 3 Seasonal Arctic atmospheric river (AR) frequency trends due to dynamic contribution in ERA5. a-b, winter (December-February) and summer (June-August) Arctic AR frequency trends due to dynamic contribution. **c**, 850 mb zonal wind trend in winter. **d**, 850 mb meridional wind trend in summer. Note that the wind trends shown in **c** and **d** are the seasonal mean wind trends. Thus, their patterns are not dependent on the AR detection algorithm used. Stippled areas indicate trends are significant at the 0.05 level based on the Student's t-test.

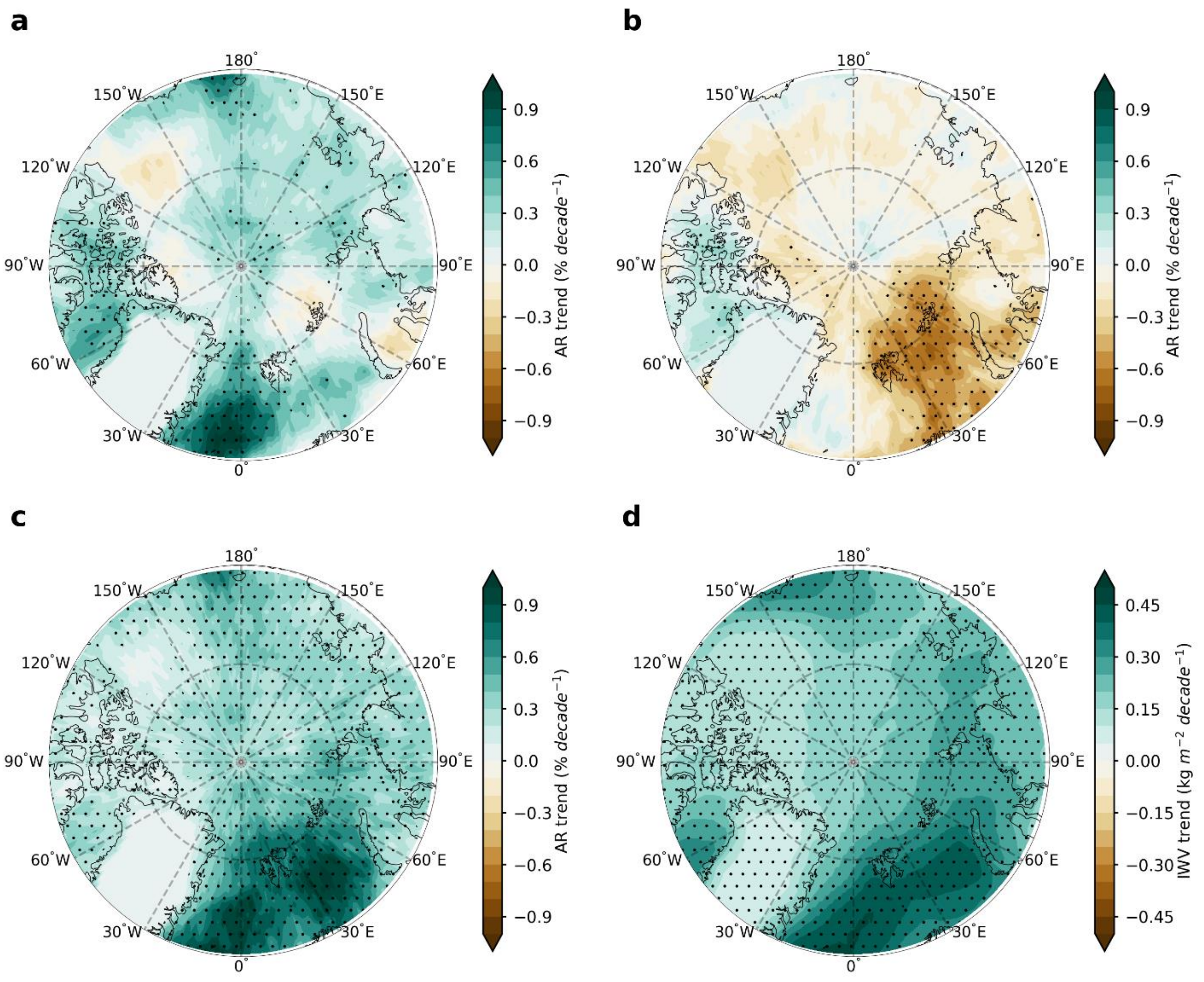

Supplementary Fig. 4 Atmospheric river (AR) trends over the Arctic in MERRA-2. a, Arctic AR frequency trend from 1981 to 2021. The dynamical contribution and thermodynamical contribution are shown in **b** and **c**, respectively. **d**, observed trends in column-integrated water vapor (IWV) from 1981 to 2021. Stippled areas in **a**, **b**, **c**, and **d** indicate trends are significant at the 0.05 level based on the Student's t-test.

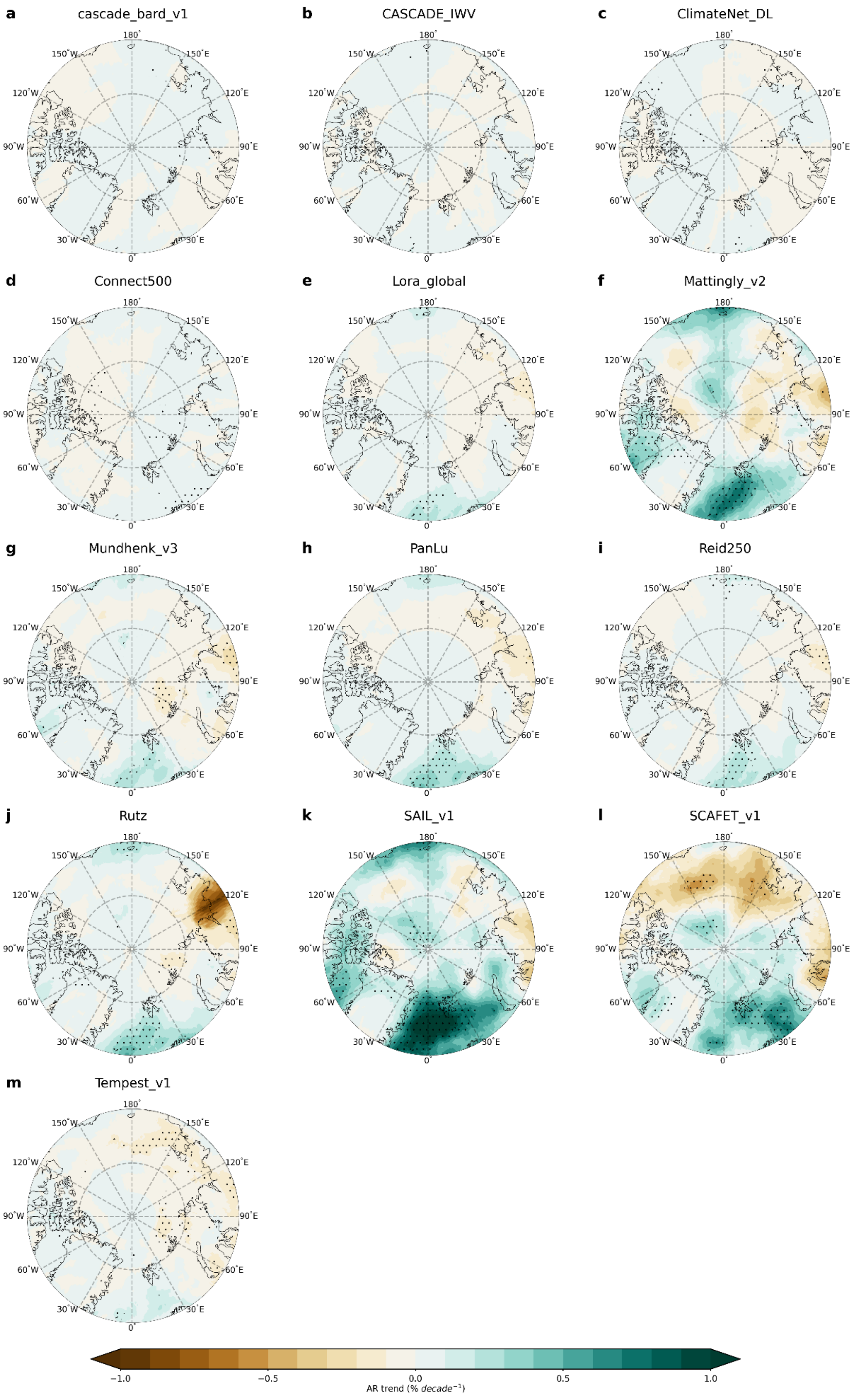

a cascade_bard_v1
b CASCADE_IWV
c ClimateNet_DL
d Connect500
e Lora_global
f Mattingly_v2
g Mundhenk_v3
h PanLu
i Reid250
j Rutz
k SAIL_v1
l SCAFET_v1
m Tempest_v1
180°
150°W
150°E
120°W
120°E
90°W
90°E
60°W
60°E
30°W
30°E
0°
−1.0
−0.5
0.0
0.5
1.0
AR trend (% decade^{-1})

Supplementary Fig. 5 Arctic Atmospheric river (AR) trends in ARTMIP. Arctic annual AR frequency trends from datasets based on various AR detection algorithms that participated in ARTMIP. These trends are based on MERRA-2 3-hourly data from 1980 to 201. Stippled areas indicate trends that are significant at the 0.05 level based on the Student's t-test. See https://www.cgd.ucar.edu/projects/artmip/algorithms for more details on the algorithms used.

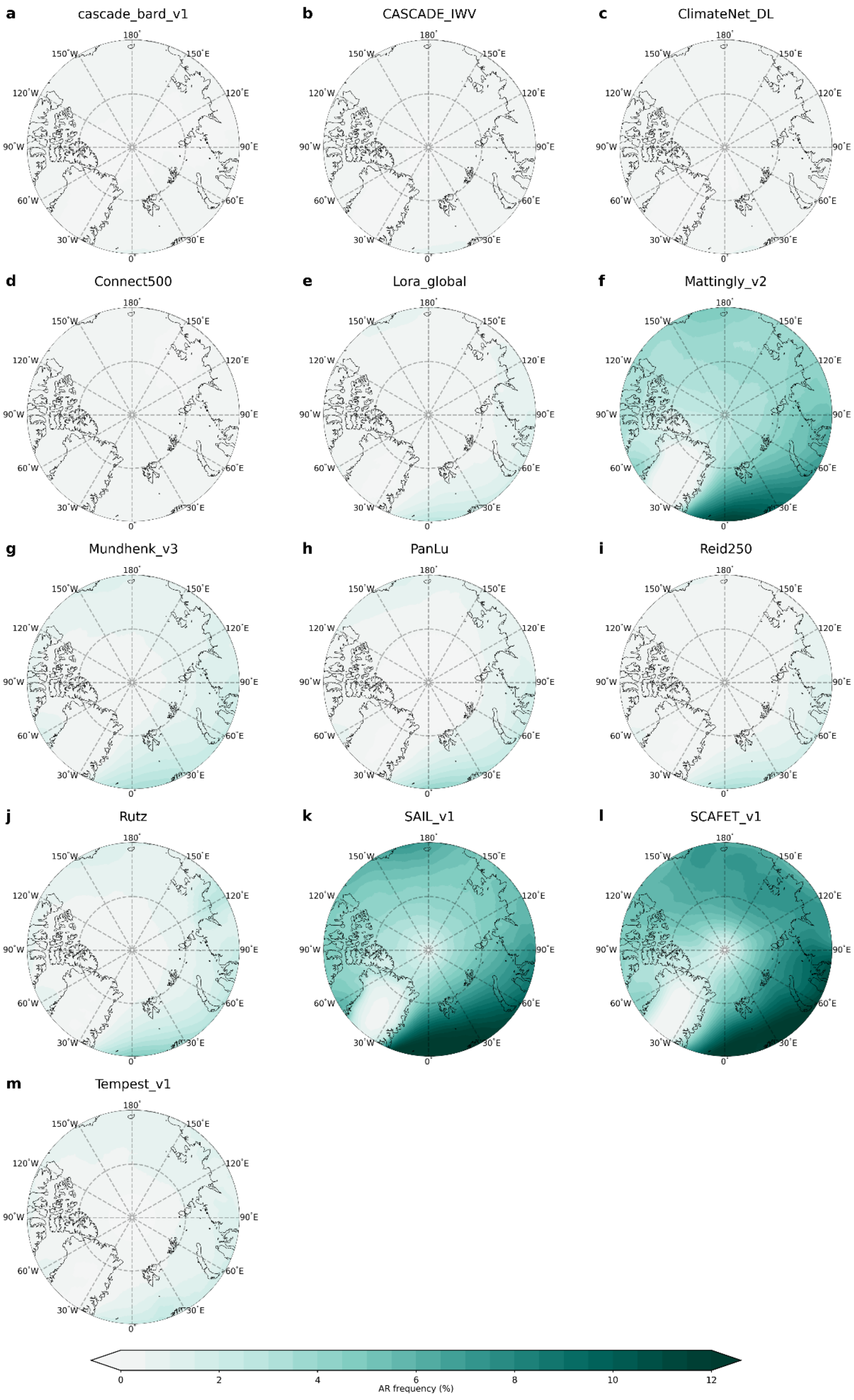

a cascade_bard_v1
b CASCADE_IWV
c ClimateNet_DL
d Connect500
e Lora_global
f Mattingly_v2
g Mundhenk_v3
h PanLu
i Reid250
j Rutz
k SAIL_v1
l SCAFET_v1
m Tempest_v1
180°
150°W
150°E
120°W
120°E
90°W
90°E
60°W
60°E
30°W
30°E
0°
0
2
4
6
8
10
12
AR frequency (%)

Supplementary Fig. 6 Atmospheric river (AR) climatology. Annual mean AR frequency (1980–2016) over the Arctic in various AR detection algorithms that participate in ARTMIP.

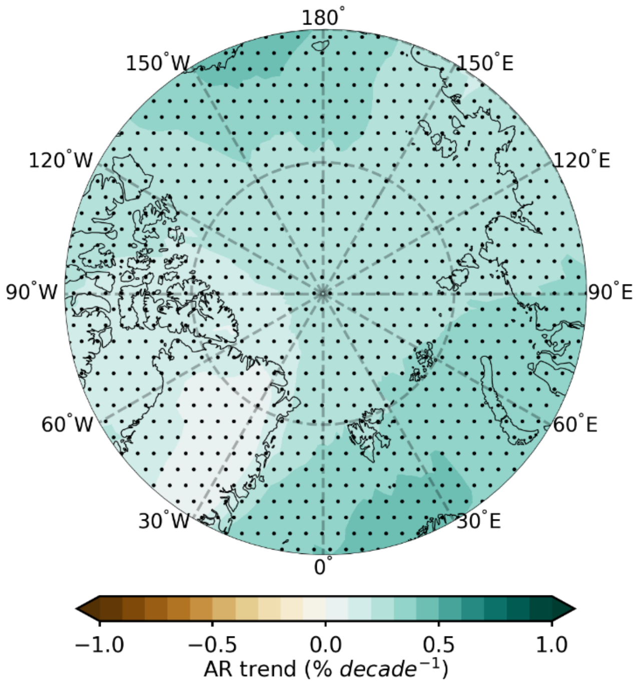

Supplementary Fig. 7 Ensemble mean atmospheric river (AR) frequency trend over the Arctic in CMIP6. The multi-model ensemble mean of Arctic AR frequency trend based on 23 CMIP6 model simulations. Stippled areas indicate trends are significant at the 0.05 level based on the Student's t-test.

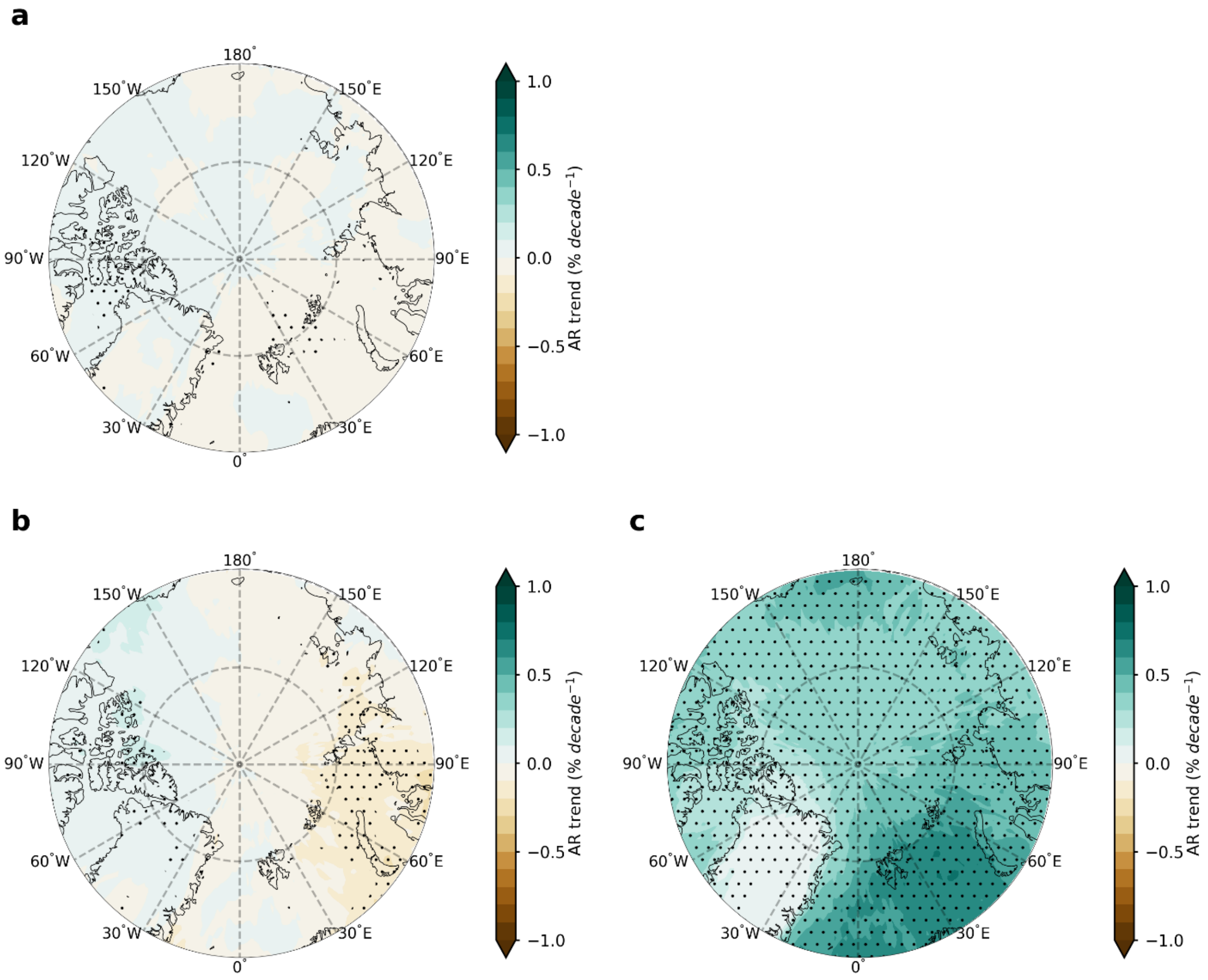

Supplementary Fig. 8 Decomposition of the atmospheric river (AR) trends in LENS2 and GOGA. a, Ensemble mean Arctic AR frequency trend in LENS2 due to dynamical changes. **b**, Ensemble mean Arctic AR frequency trend in GOGA due to dynamical changes. **c**, Same as **b**, but for the trend due to thermodynamical changes. Stippled areas indicate trends are significant at the 0.05 level based on the Student's t-test.

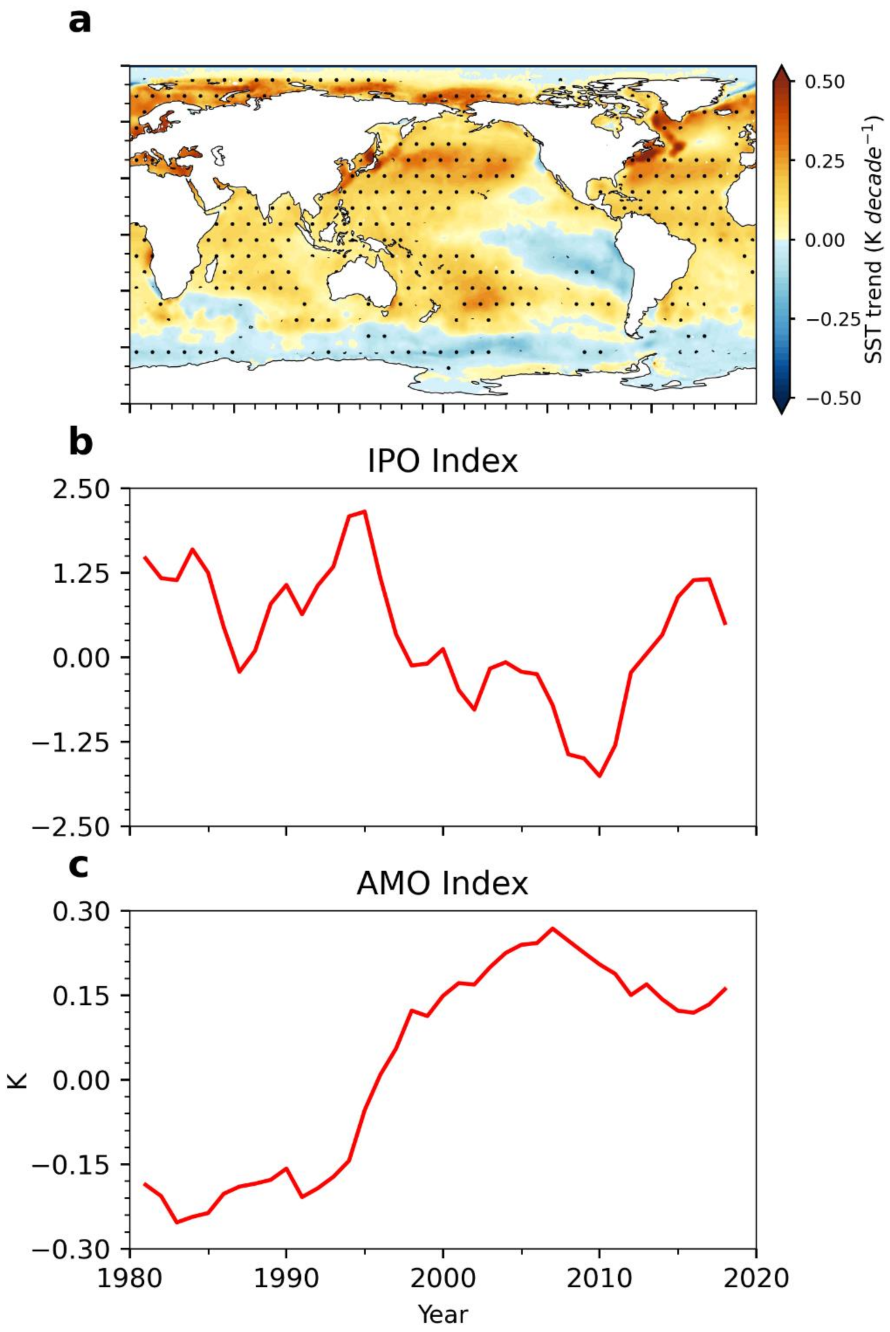

Supplementary Fig. 9 Observed Interdecadal Pacific Oscillation (IPO) and Atlantic Multidecadal Oscillation (AMO) index. a, Observed sea surface temperature (SST) trends from 1981 to 2021 based on the HadSST data. Stippled areas indicate trends are significant at the 0.05 level based on the Student's t-test. **b**, Temporal evolution of the observed IPO index. **c**, Temporal evolution of the observed AMO index.

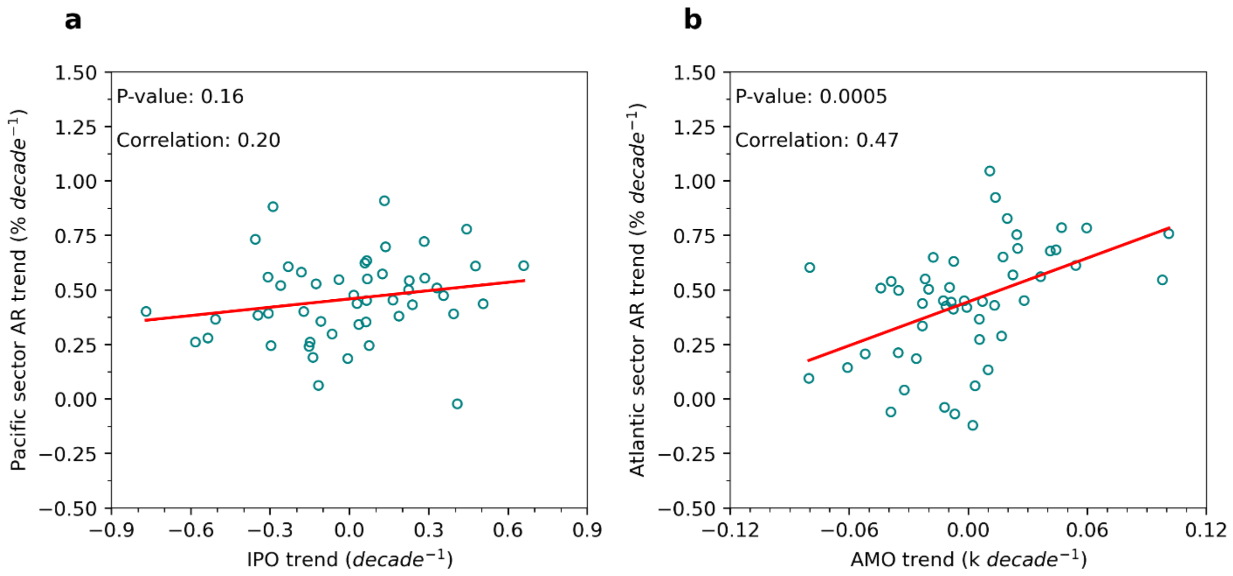

Supplementary Fig. 10 Relationship between the Pacific/Atlantic sector mean atmospheric river (AR) frequency trends and the Interdecadal Pacific Oscillation (IPO)/Atlantic Multidecadal Oscillation (AMO) trends. a, Scatterplots between the mean AR frequency trends over the Pacific sector and the IPO trends, where the red lines show the regression of data points for 50 members of LENS2. **b** same as in **a**, but for the AR trends over the Atlantic sector and AMO trends.

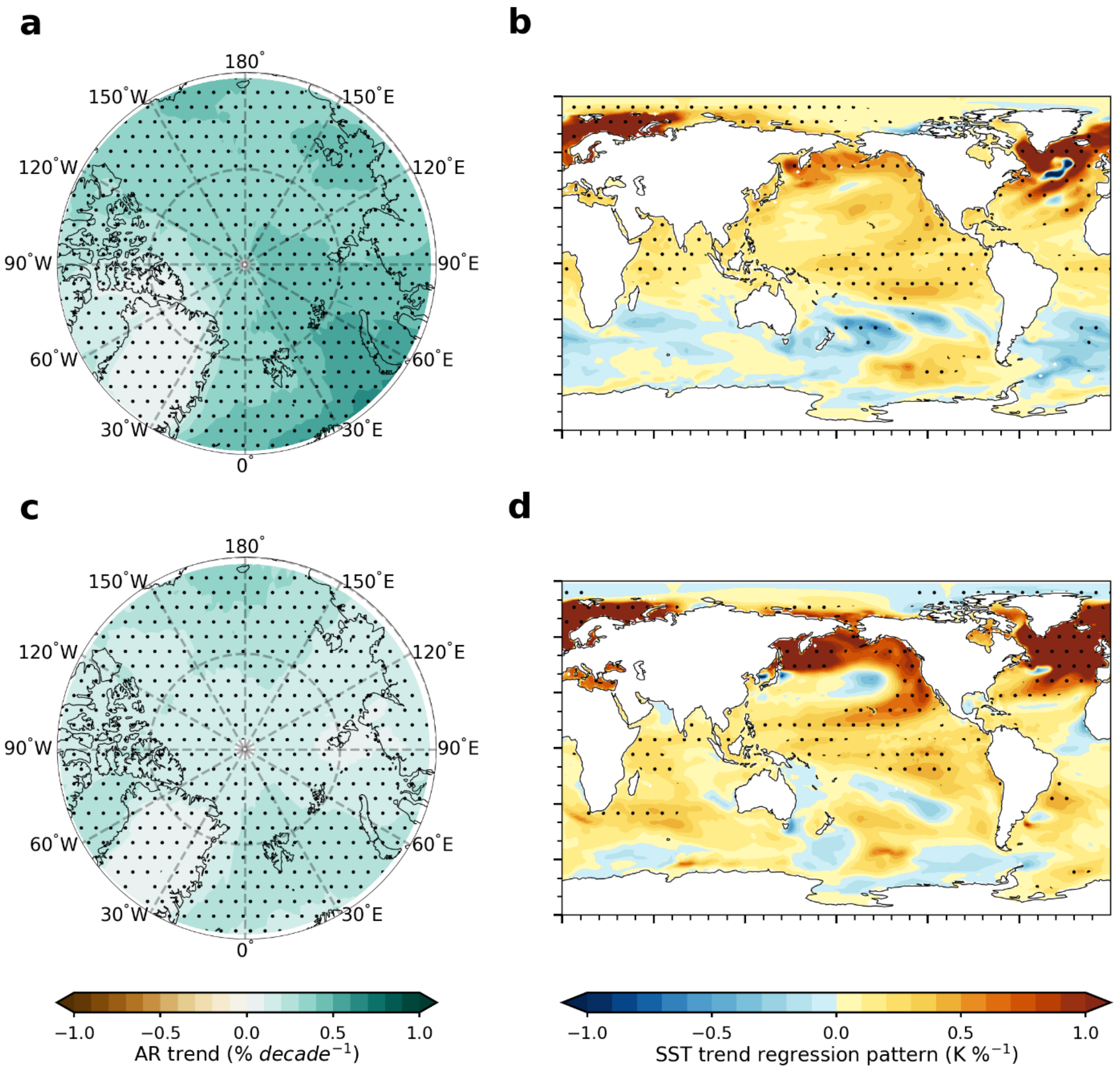

Supplementary Fig. 11 Intermember regressions in the ACCESS and CNRM ensembles. a, ensemble mean Arctic atmospheric river (AR) trend from 1981 to 2021 in the ACCESS ensemble. **b**, Intermember regression between sea surface temperature (SST) trend and Arctic mean AR frequency trend in the ACCESS ensemble. **c-d**, Same as **a-b**, but for the CNRM ensemble. The results in **c-d** are based on data from 1979 to 2014. Stippled areas indicate trends or regressions are significant at the 0.05 level based on the Student's t-test.

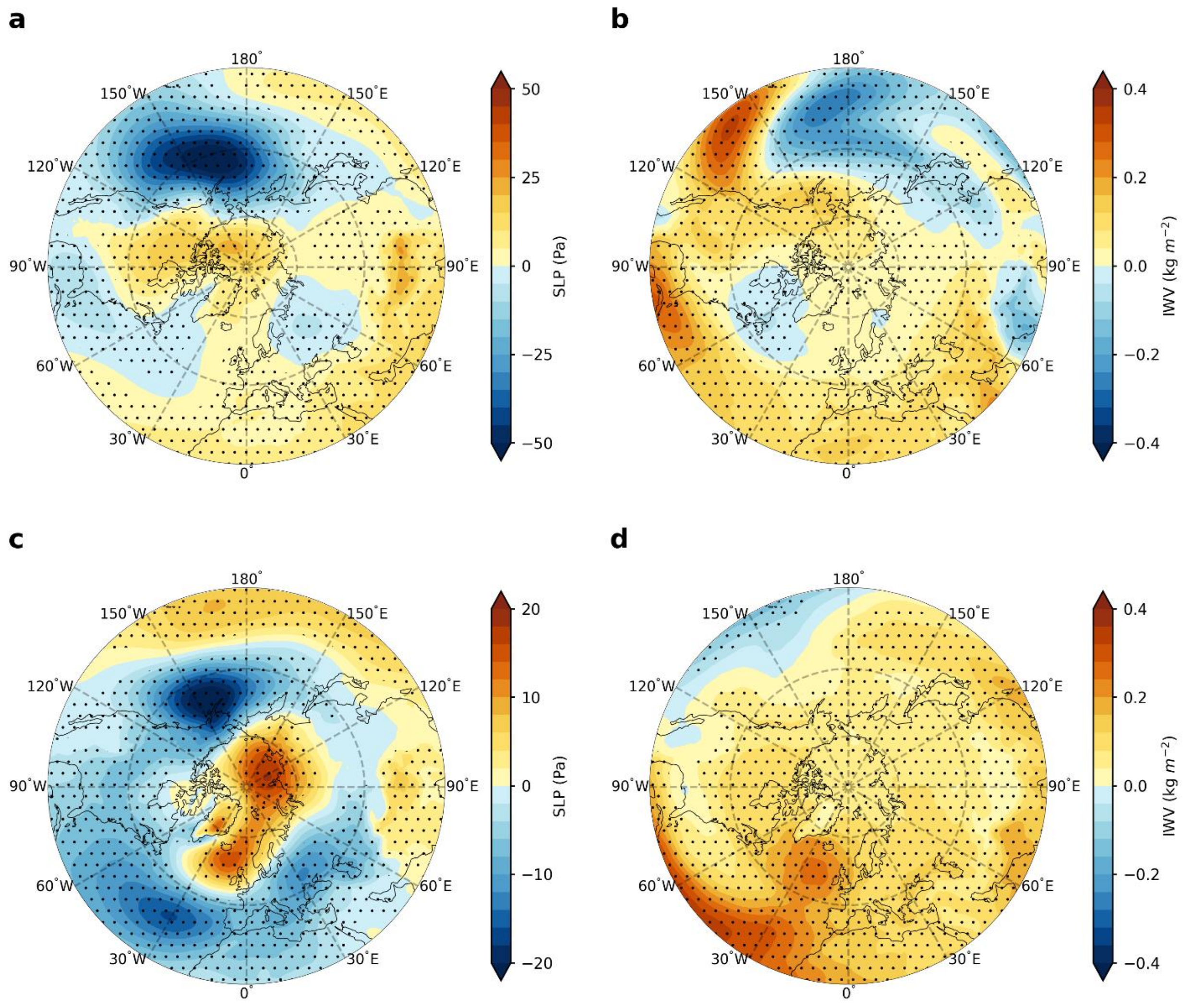

Supplementary Fig. 12 Sea level pressure (SLP) and column-integrated water vapor (IWV) regression patterns associated with Interdecadal Pacific Oscillation (IPO) and Atlantic Multidecadal Oscillation (AMO). a and **c**, Ensemble mean SLP patterns associated with the IPO and AMO, respectively, in LENS2. **b** and **d**, Same as **a** and **c**, but for the IWV. Regressions are based on data from 1979 to 2100. Stippled areas indicate the regression anomalies are significant at the 0.05 level based on the Student's t-test. Note that the range of the color bar in **c** is different from that in **a** for visualization purpose.

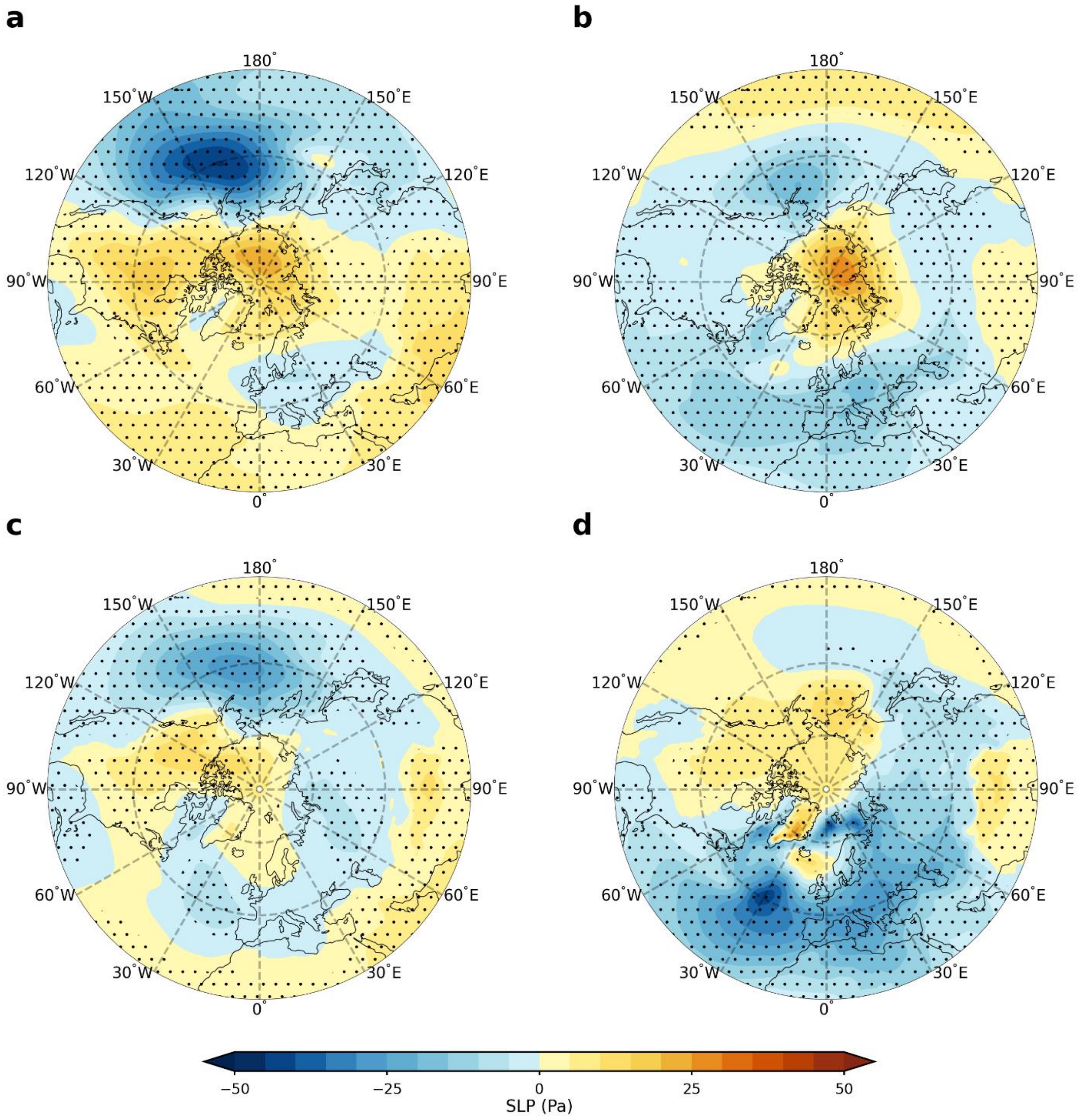

Supplementary Fig. 13 Sea level pressure (SLP) regression patterns associated with the Interdecadal Pacific Oscillation (IPO) and Atlantic Multidecadal Oscillation (AMO) in the ACCESS and CNRM ensembles. a-b, Ensemble mean SLP patterns associated with the IPO and AMO in the ACCESS ensemble. **c-d**, Same as **a-b**, but for the CNRM ensemble. The regressions for ACCESS ensemble are based on data from 1979 to 2100, but from 1850 to 2014 for CNRM ensemble. Stippled areas indicate regression anomalies are significant at the 0.05 level based on the Student's t-test.

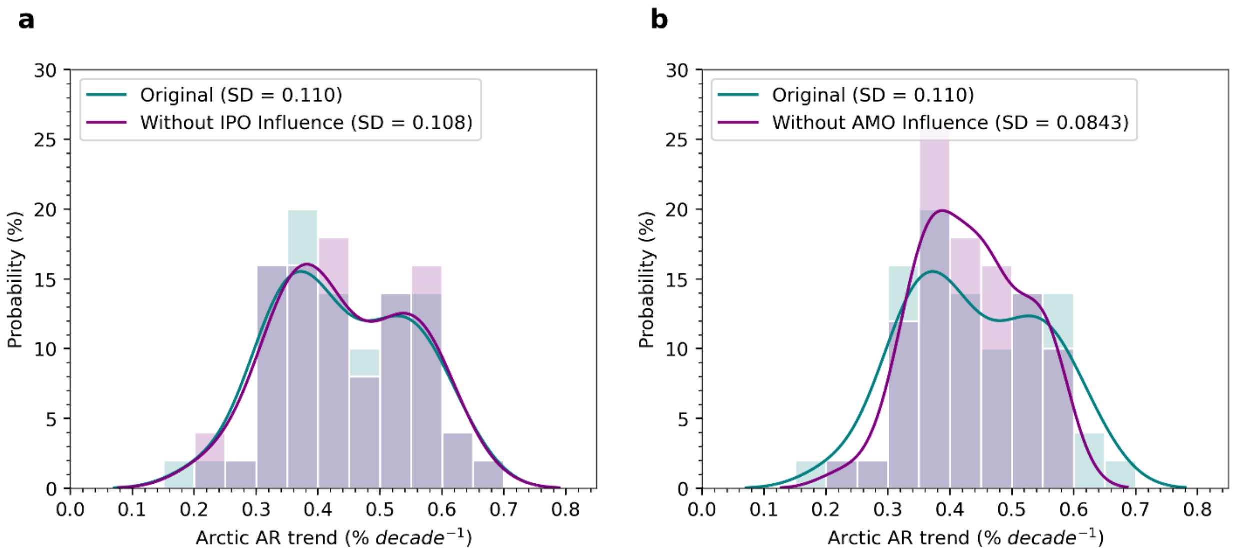

Supplementary Fig. 14 Influence of the Interdecadal Pacific Oscillation (IPO) or Atlantic Multidecadal Oscillation (AMO) on uncertainties in near-future Arctic mean atmospheric river (AR) frequency trends. Histograms and the kernel density estimates of the near-future Arctic mean AR frequency trends are shown in bars and lines, respectively. The teal bars and line represent the original Arctic mean AR frequency trends. The purple bars and line denote the Arctic mean AR frequency trends without the influences of IPO and AMO. **a**, The influence of the IPO. **b**, The influence of the AMO. Note that the gray bars indicate an overlap between purple and teal.

Table S1. Information of the 23 coupled models used in the CMIP6 multi-model ensemble analysis of Arctic atmospheric rivers

Model	Institute	Horizontal grids (lat × lon)
ACCESS-CM2	CSIRO-ARCCSS	145 × 192
BCC-ESM1	BCC	64 × 128
CanESM5	CCCma	64 × 128
CESM2-WACCM	NCAR	192 × 288
CMCC-CM2-SR5	CMCC	192 × 288
CMCC-ESM2	CMCC	192 × 288
EC-Earth3	EC-Earth-Consortium	256 × 512
EC-Earth3-Veg	EC-Earth-Consortium	256 × 512
EC-Earth3-Veg-LR	EC-Earth-Consortium	160 × 320
FGOALS-g3	CAS	80 × 180
IITM-ESM	CCCR-IITM	94 × 192
INM-CM4-8	INM	120 × 180
INM-CM5-0	INM	120 × 180
IPSL-CM5A2-INCA	IPSL	96 × 96
IPSL-CM6A-LR	IPSL	143 × 144
MIROC6	MIROC	128 × 256
MPI-ESM-1-2-HAM	HAMMOZ-Consortium	96 × 192
MPI-ESM1-2-HR	MPI-M	192 × 384
MPI-ESM1-2-LR	MPI-M	96 × 192
MRI-ESM2-0	MRI	160 × 320
NorESM2-LM	NCC	96 × 144
NorESM2-MM	NCC	192 × 288
TaiESM1	AS-RCEC	192 × 288
